# Using a theatre-based programme to prevent gender-based violence: evidence from Australia

**DOI:** 10.1093/heapro/daac025

**Published:** 2022-03-31

**Authors:** Beth R Crisp, Ann Taket

**Affiliations:** School of Health and Social Development, Deakin University, Waterfront Campus, Locked Bag 20001, Geelong, VIC 3220, Australia

**Keywords:** violence against women, theatre-based programmes, bystander interventions, Australia

## Abstract

Bystander interventions play an important contribution to efforts to prevent violence against women and arts-based interventions have been effective as part of a range of health promotion programmes. *You the Man* is a theatre-based programme, which contributes to violence prevention efforts in community settings. Requiring a single actor and minimal props, the programme consists of a 30–35 min play about intimate partner violence and sexual assault followed by a moderated post-performance panel discussion including staff from local support agencies. Although it has received positive feedback in a range of settings, the only previously published evaluation involved establishing short and long-term impacts on American high school students. This article examines the short-term impacts from attending *You the Man* events on a different audience, i.e. Australian adults. Anonymous online surveys conducted prior and 4 weeks after events were completed by 41 participants of whom 29 were female and 12 were male, three-quarters of whom were aged between 18 and 49, and who attended events at university campuses (46.3%), in workplaces (34.1%), at sporting clubs (12.2%) and community centres (7.3%). At follow up, participants regarded the severity of abusive and coercive behaviours as being higher than at baseline, their capacity to intervene as a bystander had increased, as had the number of sources of support they would recommend to someone experiencing gender-based violence. Hence, theatre-based programmes can positively affect attitudes in relation to gender-based violence, increase bystander knowledge about safe ways to intervene and positively affect intended bystander intervention.

## INTRODUCTION

Violence against women is a global problem (United Nations Statistics Division, 2021). The World Health Organization (WHO) has estimated that more than one-third (35%) of women worldwide have experienced either physical and/or sexual intimate partner violence or non-partner sexual violence (WHO, 2013). This represents not only a fundamental violation of women’s human rights but also a significant public health problem (Webster *et al.*, 2018).

Although it has been claimed that ‘Australia is among world leaders in its efforts to reduce violence against women and to promote gender equality and respect’ [(Webster *et al.*, 2018), p. 18], further efforts to prevent gender-based violence are a national priority in terms of both policy and practice (Our Watch, 2020). A 2016 national survey found that the majority (53%) of Australian women reported having been sexually harassed at least once in their lifetime. The same survey found that almost one-quarter (23%) of Australian women aged 18 and over reported having experienced violence by an intimate partner, involving physical and/or emotional abuse. For women reported having been sexually assaulted by a male, this was usually (87%) by a person known to them. More than half of these sexual assaults occurred in either the woman’s own home (40%) or that of the perpetrator (17%), with the remainder (43%) occurring elsewhere (Australian Bureau of Statistics, 2017).

While physical abuse has been widely recognized in Australia as a form of domestic violence over the last two decades, forms of emotional abuse, such as controlling who a woman spends time with or her use of money, have increased significantly but are still less likely to be regarded as forms of abuse (Webster *et al.*, 2018). Changing social norms is a key component in achieving effective prevention of violence (Cislaghi *et al.*, 2019; Orchowski *et al.*, 2020) and addressing gender inequality is integral to Australian policy and practice aimed at preventing gender-based violence (Our Watch *et al.*, 2015).

Increasing awareness of the diversity of abusive behaviours increases the likelihood of bystanders intervening on behalf of a woman. Although bystanders may be strangers who directly intervene in an incident of abuse, which they are observing (Carlson, 2008), bystander interventions also include encouraging individuals who are perceived as being in danger to take action for themselves (Jouriles *et al.*, 2018) and/or take other actions, which do not involve direct contact with a person they believe to be perpetrating abuse (Carlson, 2008). However, even if they notice a situation and determine that intervention is required, bystanders need to be aware of what actions they can take and have confidence in their capacity to take action (Latané and Darley, 1970). Hence, developing ‘belief in one’s ability to de-escalate a violent situation effectively’ [(Jouriles *et al.*, 2018), p. 457] is a core component of bystander interventions.

Internationally there has been widespread acceptance of the proposition that tackling gender inequity is a critical component to preventing violence against women (United Nations Statistics Division, 2021). Although this is being challenged in some countries [e.g. (Öhman *et al.*, 2020; Wemrell *et al.*, 2021)], the Australian policy context at the time this research was undertaken was that programmes, which effectively tackle gender-based violence are those which recognize that violence against women has been enabled by cultures sanctioning unequal relationships between men and women (VicHealth, 2007; Our Watch *et al.*, 2015). Moreover, such programmes are premised on an understanding that violence against women is not accidental but a logical outcome of factors, such as entrenched sexism and gender discrimination (Savigny, 2020). However, these are not necessarily seen as being serious problems by many in the Australian community (Jenkins, 2017). Therefore, bystander interventions, which challenge sexism and discrimination, have been proposed as an approach to preventing of gender-based violence (Powell, 2012).

The programme, reported in this article, draws on an evidence base provided by a wide range of research into violence prevention, bystander interventions and arts-based methods in health promotion [e.g. (Iverson, 2006; Bowles and Nadon, 2013; Iverson and Seher, 2014; Taket, 2020)]. Berkowitz (Berkowitz, 2001, 2004) summarized the characteristics of effective prevention programmes, which in the case of promoting gender equity need to be:

- *comprehensive*, in that they address and involve all relevant community members and systems.
- *intensive*, in that they offer learning opportunities that are interactive, involve active participation, are sustained over time and have multiple points of contact with reinforcing messages.
- addressing *cognitive, affectiveand behavioural domains*: what people know, how they feel and how they behave.
- *relevant to the audience*. They are tailored to the characteristics of the participants and acknowledge the special needs and concerns of particular communities. They focus on peer-related variables, use peers in leadership roles, and emphasize the relationship of sexual assault to other issues.
- offering *positive messages* which build on men’s values and predisposition to act in a positive manner

*You the Man* (*YTM*) is a theatre-based education programme for bystander engagement and violence prevention, and is an example of the increasing of arts-based methods for the promotion of health and wellbeing (Fraser and al Sayah, 2011). It is a brief intervention, consisting of a 30–35 min play about intimate partner violence performed by a single actor, plus a moderated post-performance panel discussion. The script of the play remains unchanged from performance to performance, but relevance is addressed by the format of the post-performance discussion being tailored to the local setting, circumstances and other violence prevention activities happening locally (Plourde *et al.*, 2016).

The programme has been carefully constructed in collaboration with a wide range of people working in the field ‘to ensure that the program: serves the survivors/victims of sexual assault and domestic violence; strengthens each community in which it is delivered; gives good information without replicating oppression; [and] transcends stereotypes’ [(Plourde *et al.*, 2014), p. 378]. In particular, the play seeks to avoid victim blaming and instead proposes that addressing violence and abuse is a shared responsibility, including bystanders (Plourde *et al.*, 2014). The *YTM* programme is grounded by Freirean pedagogy (Freire, 1993) and Theatre of the Oppressed principles (Boal, 1997), with the aim of the event being to galvanize stakeholders and leave the community in stronger position. Moreover, it also encourages viewers to act on their suspicions that a situation is abusive.

*YTM* is designed for delivery to mixed or single gender groups of participants and makes the assumption that those in the audience have the capacity for empathy and that, like the various characters in the play would want to do what is possible to prevent violence from occurring. Full scripts for both the American and Australian versions are available (Plourde, 2018) as well as details of the plot summary and characters (Plourde *et al.*, 2014, 2016).

The delivery style is direct address—each of the characters performed by the actor is engaged in a conversation with the audience, in order to maximize their engagement (Thatcher, 2011). The goal was particularly to reach the male bystanders, the ‘mass in the middle’ who are not perpetrators and to activate them as allies by using humour and headlines to honour the complexity and seriousness of the issues involved [see also (Kamis and Iverson, 2016)]. The various characters in the play speak to many different possible bystander situations, and encourage the audience (males and females) to consider what they would do in these situations.

Although conversational in style, the play is not interactive. Despite much of the literature on theatre in education arguing that participation enhances learning, the empirical evidence is equivocal (Heard *et al.*, 2020). For example, Ahrens *et al.* (Ahrens *et al.*, 2011) found there was no significant difference on self-reported intention to intervene according to degree of participation in a performance and concerns about lack of rigour in the evaluation of theatre-based programmes have been raised (Taboada *et al.*, 2016; Heard *et al.*, 2020). In the current context, however, there were strong arguments for a performance, which utilized direct address and was non-interactive. These included a dearth of men in positive bystander roles in the media compared to representations of men as perpetrators of violence against women. The fixed script of *YTM* ensures the portrayal of men as bystanders is not derailed by audience members seeking to change the narrative through debates with the actor while the play is being performed. Control of the script is also necessary as along with the presence of the panel for the subsequent discussion, this creates a level of safety for the audience, which may include victims and survivors of domestic violence and/or sexual assault (Plourde *et al.*, 2014).

Organizations hosting *YTM* are required to provide a moderator to introduce the play and facilitate the panel discussion. It is important that this person is perceived as a credible leader within the organization and does not need to be an expert in violence prevention. Panel members present for this discussion are drawn from local support agencies involved in domestic violence and sexual assault, including specialist services, police, health services and counsellors. The discussion serves to introduce people to the local resources that are available. A comprehensive resource pack is provided to assist the local organizer(s) to set up the programme for their particular setting.

The play is very carefully constructed to provide accurate and critical information, highlight community services, and have high artistic merit while holding simplified production requirements. This requires ‘clear criteria [that] ensures that capable artists are recruited and high-quality artworks produced’ [(Ninnes and Koens, 2019), p. 7]. Hence, a professional theatre director and professional actors were recruited to direct and perform *YTM*. Moreover,

Professional actors are trained in delivery, and can manage audiences who can be unruly or disrespectful more adeptly than an amateur … and the high artistic merit lessens the risk of public ridicule of the topic [(Plourde *et al.*, 2016), p. 233].

In each performance, a single male actor plays six contrasting male characters, none of which are perpetrators. Furthermore, the key concerns of each character are issues, which transcend national borders, race, class and other socio-economic factors (United Nations Statistics Division, 2021). The play shows the impact of violence and models different forms of supportive bystander responses, including those listed in Table 4, which family and friends of people experiencing intimate partner violence could consider implementing. During the development phase, the play’s author was encouraged to use male voices in order to engage with young males, who often failed to engage with violence prevention messages, which were typically presented by females. It is also important for females to see that violence against women is not something their gender has to tackle on its own (Plourde *et al.*, 2014).

**Table 4:** Utilization of bystander strategies with friends who have experienced abuse/coercion in relationships

Behaviour	Baseline %	Follow-up %
Told them to leave person	44.8	51.6
Sought advice from a friend	13.8	25.8
Supported them and listened when they needed it	82.8	71.0
Recommended they called a support or crisis line	55.2	41.9
Called a support or crisis hotline	10.3	12.9
Talked to a health professional	10.3	6.5
Told that person’s parent or other family member	3.4	0.0
Other	0.0	12.9
Nothing	6.9	3.2
*n*	29	31

In addition to using a single actor, other requirements identified at the development phase were that the play used a minimum of props, had low or no-technical requirements and could be performed easily in a wide variety of venues, keeping the cost of the programme to a minimum and maximizing ease with which the programme could be brought into different communities and locales. The requirements in terms of staging involve no special lighting or sound effects, making it possible to deliver the programme in a wide variety of different spaces. Venues are required to provide minimal props (table and chair) and the actor provides other props carried in a single portable bag. These features means that it is possible for the programme to be delivered in the relevant local setting for the community to which in is being delivered: in a school hall, in the club rooms of a sports club, in a meeting room in a community centre or workplace, in a classroom or hall in a tertiary education institution.

*YTM* was originally developed in the USA, and has been in use there since 2002. An Australian version of the programme was developed over the period May to November 2013. The core team involved in the work was drawn from the Faculties of Health and Arts and Education at Deakin University, with active participation in the development process by a diverse group of people working in the violence prevention field from across the state of Victoria. A series of informal readings was held with a range of experts in the field of violence prevention to confirm the applicability and appropriateness of its contents. The changes made were predominantly concerned with replacing American colloquial language with Australian equivalents, changing the names of two characters and changing the sporting code from basketball to Australian rules football. A highly experienced director was engaged and by the end of September 2013 she had cast two actors to join the team. The actors were rehearsed in October and early November and the development phase ended with four ‘preview’ deliveries in November 2013 (Plourde *et al.*, 2014; Plourde, 2018). Since March 2014, the programme has been delivered in a range of different settings across the state of Victoria.

Bystander programmes are becoming widely used in efforts to prevent gender-based violence, as the evidence base for such interventions is growing [e.g. (Flood, 2011; Powell, 2012; Storer *et al.*, 2016; Taket and Crisp, 2017; Jouriles *et al.*, 2018; Crooks *et al.*, 2019)]. *YTM* has been evaluated and found to be effective amongst high school students in the USA, with significant effects found in student cohorts 3 years after being exposed to *YTM* (Plourde *et al.*, 2016). However, there have been no evaluations of the effectiveness of YTM as an intervention with adults, or with audiences in other countries.

## METHOD

This purpose of this investigation is to explore the efficacy of *YTM* as a bystander intervention for Australian adults.

### Participants

The participants in this study were 41 adults aged 18 and over who had seen *YTM* delivered in Victoria, Australia in 2014 who had completed surveys both prior to, and after, attending the performance. Two participants, one male and one female, reported having seen *YTM* previously on YouTube, which would have been the American version of the play (a performance of the American version of the play, along with the subsequent moderated discussion is available at https://www.youtube.com/watch?v=_hf3F8q3EJg). The majority (70.7%) of participants were female but almost one-third were male (29.3%). While almost one-third (31.7%) were aged 18–29, almost one-quarter (22.0%) were aged 30–39 and the same number (22.0%) were aged 40–49. There were also some participants aged 50–59 (14.6%) and aged 60 and over (2.4%). The age of three participants (7.3%) is unknown. The age range and gender mix of range of participants is reflective of the different demographics of the settings in which the play was performed which were university campuses (46.3%), workplaces (34.1%), sporting clubs (12. 2%) and community centres (7.3%).

### Data

Previous research into bystander interventions has typically utilized multiple measures associated with understandings of the severity of problems, confidence to take action, supports offered and/or utilization of bystander behaviours (Storer *et al.*, 2016). Although the design of Plourde *et al.*’s (Plourde *et al.*, 2016) study differed from the current research in that it involved a pre-test, post-test shortly after the YTM event and follow-up surveys in both of the two subsequent years, each wave collected data on the same measures. In consultation with Plourde, these measures were adapted to reflect Australian language and the contexts in which YTM was presented and these data collected both prior and following the performance. With the exception of the baseline data, the American *YTM* study also collected information from participants about their learning at each point in time. This study collected this data at follow-up only. Each of these data types are explained below.

#### Perceived severity of behaviours

Participants were asked to rate how serious a problem they consider nine forms of abuse and controlling behaviours to be when they are directed towards a person one is in a relationship with. Answers were on a six-point scale from ‘Not at all serious’ (scored as 1) to ‘Very serious’ (scored as 6).

#### Confidence to take action

Participants answered how able they feel to take action to protect themselves or others, and whether they have the tools and resources to take action. Although what actions or behaviours participants were being asked about were not specifically stated, questions about perceived behavioural control were preceded by questions about abusive coercive behaviours, such as those noted in Table 1. At follow up, participants were also asked the extent to which *YTM* had contributed to their perceptions. Answers to each question were on a five-point scale from ‘Definitely not’ (scored as 1) to ‘Definitely’ (scored as 5).

**Table 1:** Perceived severity of behaviours at baseline and follow-up

Behaviour	Baseline rated very serious %	Follow-up rated very serious %	Baseline mean rating	Follow-up mean rating	Significance of paired samples *T*-test	*n*
Puts down, insults or calls the person names	10.0	20.0	4.35	4.78	<0.001	40
Tells the person they cannot do certain things	12.2	24.4	4.34	4.98	<0.001	41
Tells the person they cannot go certain places	14.6	26.8	4.59	5.02	<0.001	41
Tells the person they cannot talk to certain people	17.1	34.1	4.83	5.17	<0.01	41
Forces the person to engage in sexual activities	73.2	90.2	5.68	5.88	<0.01	41
Threatens the person	77.5	75.0	5.73	5.75	n.s.	40
Hits, punches, shoves or physically hurts the person	87.8	92.7	5.85	5.93	n.s.	41
Feels unsafe with or does not trust the person	68.3	75.6	5.66	5.68	n.s.	41
Is absent from school/work	78.0	76.0	5.76	5.83	n.s.	41

#### Available sources of support

Participants were asked who they would advise a friend or peer who disclosed they were in an abusive relationship to talk with. The online survey included a list of options, which included both formal and informal supports. The total number of responses provided a measure of available supports that could be compared over time.

#### Utilization of bystander behaviours

Participants who stated they knew someone in an abusive relationship were asked to state what they had done in response. The online survey included a list of options and the total number of responses provided a measure of bystander actions that could be compared between baseline and follow up.

#### Learning from YTM

In the follow-up survey, prior to answering questions in respect of the above measures, participants were asked what they had learnt from attending a YTM event. This was a free-text field in which participants could write whatever they wanted to in response to this question.

### Procedure

During the period when this research was conducted, local organizers sent out an invitation to participate in an evaluation of YTM in Australia prior to the event. This was sent to everyone who had registered to attend the YTM event and included a letter from the researchers, which included the following text:

This invitation has been sent to you by the local organizer of the *You the Man* program, and is being sent to all those who have registered their attendance for the forthcoming program.

As you may know, the *You the Man* Program is a theatre-based program for bystander engagement and violence prevention. It has been successfully used in the United States for over 10 years, with positive effects on knowledge attitudes and behaviour in the short, medium and longer term. Now that we have an Australian version of the program being used in Victoria, we think it is important to investigate the short-term effects of the Program within the settings it is now being used. This study will look at its short term effects.

The invitation also included a plain language statement, and instructions for how to access the online survey. Contact details (telephone and email) for the research team were also provided should individuals wish to seek any additional information before deciding whether or not to participate. Baseline surveys, completed prior to the programme delivery, were completed anonymously and completing the survey was taken as indicating consent to participate in that part of the research. At 4–6 weeks after the event, local organizers sent the invitation to the post-programme survey to the same individuals as before, with no knowledge as to who had completed the first survey.

Participants who completed the surveys prior and after the programme entered the results into an online survey created using Survey Monkey. Thus, participants provided survey responses directly to the researchers. In order to enable baseline and follow-up data from participants to be linked, participants were asked to create an identification code comprising the first letter of their name and the last four digits of their telephone number.

### Ethical concerns

Ethical approval for the research was granted by Deakin University (approval HEAG-H 139_2013).

As the subject matter of the performance and subsequent discussion include intimate partner violence, domestic violence and sexual coercion, issues of safety and support are covered in some detail in the implementation guide. These include requirements for the provision of a safe room and counselling during each *YTM* programme delivery. This was available to all attendees irrespective of whether or not they were participants in the research study. In line with being a theatrical production, a play programme was provided to participants, which includes key Victorian telephone line resources for later help and assistance as well as insert on local resources produced by the local organizer. The online surveys concluded with a reminder of state-wide support resources, giving relevant helpline numbers.

### Analyses

Paired *T*-tests were used to compare baseline and follow-up data for the linear variables concerning perceived severity of behaviours, confidence to take action, the number of sources of support and the number of bystander actions taken. A thematic analysis of comments about learning was conducted.

## RESULTS

### Learning from YTM

When completing the post-performance survey, all but one of the participants claimed to have learnt something from attending *YTM*. When asked to describe what they had learnt, all but two of the remaining participants provided a description of the key learning for them, and all these descriptions were consistent with messages provided in the *YTM* programme.

A key learning was of the difference that bystanders can make if they choose to act. For example:

By being a passive bystander you are complicit and essentially condoning the behaviour/actions/attitudes of others. You can be an active bystander by speaking out and not ignoring others inappropriate behaviour etc. Also there are options and ways to assist those who are experiencing partner violence as a bystander.

Similarly,

Our actions affect more people than just ourselves. There is no such thing as an innocent bystander—if you stand by and do nothing you are taking part in the incident.

Moreover,

Someone needs to speak up in the case of abusive relationships. You cannot just ignore the matter and expect it to be handled by itself. It involves more than the lives of the individuals directly involved in the matter.

Nevertheless, it was acknowledged that it can place bystanders in an invidious position, particularly if their observations are rejected by the person(s) they believe are being subject to abuse. In such situations, it is important that other bystanders do not take the responses to someone else as a reason not to act.

Some participants reported that their understandings of what was considered abuse had expanded:

That abuse is more than just physical. Emotional abuse occurs when you ban your partner from going certain places or meeting up with certain people. Also that it is often very hard to help someone suffering from abuse as they may not admit to themselves that there is a problem.

Consequently,

Helping yourself understand will mean you can be more effective in helping others in potentially abusive relationships.

A few participants also commented that they would react differently if a disclosure of violence was made to them:

Your reaction to someone disclosing violence to you is really important and its best to just listen rather than angrily yelling, telling them to leave, asking them why they haven’t already left etc.

For at least one participant, attending the play had given them a better understanding of what help might be available should they ever find themselves in an abusive relationship:

That if I ever need helps there are lots of ways for me to get it and people to go to. Different perspectives from specialists and their way of handling issues. That I am never alone.

The extent to which these comments from individuals reflected the experience of all participants was tested by comparing pre- and follow-up data on relevant measures.

### Perceived severity of behaviours

Table 1 shows the percentage of participants who rated each of nine problematic behaviours as being very serious at both baseline and at follow up. A paired sample *T*-Test revealed mean number of problems considered very serious increased from 4.49 to 5.36 [T(38) = 3.254, *p* < 0.01]. As ratings of severity can increase but not necessarily change the percentage of participants rating a behaviour as being very serious, paired samples *T*-tests were used to determine whether to whether the overall ratings of severity increased over time. A mean increase in ratings of severity was found for the four behaviours which less than half of participants considered to be ‘very serious’ at baseline, although the number of participants who rating these behaviours as ‘very serious’ remained a minority. Of the five behaviours which the majority of participants rated as ‘very serious’ at baseline, a significant increase in the rating of severity was found only in respect of forcing a person to engage in sexual activities against their will.

### Confidence to take action

Table 2 shows that the percentage of respondents who definitely believed they (i) had the tools and resources to take action, and (ii) would do so on becoming aware of abuse being perpetrated on others, increased after seeing *YTM*. Paired samples *T*-tests revealed that participants overall ratings of perceived control in these situations significantly increased between baseline and follow up. At follow up, more than one-third (39.0%) of participants agreed that seeing *YTM* had definitely resourced them to take action against abuse, with one-quarter (24.4%) of participants sating that seeing *YTM* had definitely made a difference as to the likelihood of them taking bystander actions. However, participant’s perceived capacity to respond if they themselves were to be subjected to abuse did not alter over time. Nevertheless, one-third (37.5%) believed that having seen *YTM* had definitely contributed to their capacity to take action if ever in an abusive relationship.

**Table 2:** Perceived confidence to take action at baseline and follow-up

Action	Baseline definitely%	Follow-up definitely %	For whom *YTM* had definitely contributed %	Baseline rating	Follow-up rating	Significance of paired samples *T*-test	*n*
Able to take action on behalf of someone else	24.4	36.6	24.4	4.00	4.32	0.005	41
Able to take action on behalf of myself	65.9	65.9	37.5	4.59	4.60	n.s.	41
Have the tools and resources to take action	40.0	58.5	39.0	4.13	4.55	0.002	40

### Available sources of support

Participants were asked a hypothetical question concerning whom they would suggest as a source of help for someone they knew who was in an abusive relationship. All participants nominated at least one source of help at both baseline and follow up. The mean number of suggestions increased from 3.75 at baseline to 4.25 at follow up, and a paired samples *T*-test revealed this increase was significant [T(39) = 2.431, *p* < 0.05]. Sources of support which participants noted they would suggest are presented in Table 3.

**Table 3:** Available sources of support at baseline and follow-up

Support	Baseline %	Follow-up %
Counsellor/student welfare	31.7	40.0
Their parents or other family member	31.7	35.0
Doctor or other health professional	56.1	55.0
DV service or hotline	85.4	97.55
Sexual assault service or hotline	75.6	92.5
Police	36.6	40.0
Friends	43.9	47.5
Someone they trust	7.3	7.5
Teachers	0.0	5.0
Workplace management	2.4	2.5
Someone else	2.4	2.5
No one	0.0	0.0
*n*	41	40

### Utilization of bystander behaviours

Almost three-quarters of participants (70.7% at baseline and 75.6% at follow up) reported having had a friend who had been in an abusive relationship. Table 4 shows that almost all of these participants reported having taking one or more actions in response to a disclosure of abuse. The most frequently mentioned strategy at each time point was to listen and provide support when it was needed. The two other most frequently reported strategies were suggesting the person call a support or crisis line, and telling them to leave their partner. While there were some changes as to what strategies participants reported, the mean number of strategies reported did not significantly change [baseline = 2.31, follow up = 2.36, T(25) = 0.493, n.s.)].

## DISCUSSION

*YTM* involves the presentation of a play about partner violence followed by an expert discussion panel. Having already been found effective among American high school students in improving understandings of what constitutes abusive relationships and increasing capacity for bystanders to act (Plourde *et al.*, 2016), the data presented in this article provide evidence that *YTM* is also effective with adult audiences in Australia in a range of settings including educational institutions, workplaces, sporting clubs and community groups, although further research is required to examine the effectiveness of the intervention for people of differing ages, genders and other socio-economic indicators. This is unsurprising given that ‘the arts provide a non-confrontational and engaging medium in which to discuss and raise awareness’ of abusive relationships [(Ninnes and Koens, 2019), p. 7]. Nevertheless, as found previously, coercive behaviours are regarded as far less serious than other forms of abuse (Stark, 2012). Hence interventions with a stronger focus on coercion would complement *YTM*.

Moreover, the results concur with previous research, which has found that bystander programmes lead to increases in both willingness and confidence that participants can effectively intervene witnessing abusive relationships (Storer *et al.*, 2016). Even single sessions of providing information in community settings has been known to (i) increase the percentage of the population who believe they have a role in preventing violence and (ii) increase the number of actions individuals feel they can take to prevent family violence (Ninnes and Koens, 2019). Moreover, there was substantial coverage of issues in the Australian media at the time when this research was conducted (McDonald and Charlesworth, 2013; Easteal *et al.*, 2015) and single session interventions, such as *YTM*, can reinforce messages about violence prevention already circulating in the community (Australian Women’s Health Network, 2014). This is consistent with research evaluating the Theory of Reasoned Behavior, which has found that building perceived capacity directly leads to an increase in both behavioural intention and actually taking action (Madden *et al.*, 1992).

Interestingly, participants indicated an increase in their belief that they are able to take action on behalf someone else but not themselves. Prior to seeing *YTM*, participants felt much more confident to take action in respect to themselves than on behalf of someone else, but the post-performance data revealed a similar level of confidence. Increased confidence in being able to engage in bystander actions is consistent with the emphasis of *YTM*. Increased confidence to act on behalf of someone else is also reflected by a significant increase in the number of sources of support they were available of at the time of follow-up. This finding also suggests that theatre-based programmes, such as *YTM*, are not only changing the capacity of individuals to take bystander actions but also contributing to a changing of cultural norms around taking responsibility to prevent harm occurring within the community [see also (Heard *et al.*, 2020)].

Despite increased confidence, as with previous studies, no significant increases in actually implementing bystander behaviours was found (Storer *et al.*, 2016). While this may be due to bystanders fearing reprisals due to intervening (Carlson, 2008), another possibility is that the relatively short-time periods for follow up in most bystander studies (Jouriles *et al.*, 2018), do not provide participants with sufficient opportunity to implement their learning.

There were however a number of differences in findings between Australian adults and American school students as to the impacts of *YTM* (Plourde *et al.*, 2016). Like the American school students on whom *YTM* research has previously been undertaken, the participants in this study did not perceive a greater capacity to take action if they found themselves subjected to abuse within a relationship. However, this study found that adult Australians were both more likely to agree that they had the tools and resources to take action and perceive they would take action when aware of abuse happening to others. Furthermore, at both baseline and follow up, all of the participants in this study were able to suggest one or more sources of support, whereas at each time the American school students were surveyed, there were always some who had no suggestions.

In terms of actual behaviours, American students were most likely to tell a friend to leave an abusive relationship, whereas the Australian adults were more likely to listen to someone and support them when they needed it. Moreover, ∼20% of American students reported taking no action at each time point, whereas as very few of the current participants did this. This would at partially account for the mean number of strategies reported by the American students ranging between 1.57 and 1.71, which is considerably less than the 2.31–2.38 strategies reported by the current sample. In addition to a higher baseline, the lack of change between baseline and follow-up in respect of helping strategies employed by the Australian sample may also reflect the fact that follow-up data are restricted to 1 month after viewing *YTM*. Compared to American audiences who were followed up for more than 2 years, no change in behaviour by the Australian participants may reflect a lack of time in which to put the new knowledge into practice.

It would be easy to dismiss differences between the American high school students and Australian adults as being due to age or country differences, and while these cannot be discounted some other factors must also be considered. Firstly, the participants in the Australian study were self-selected and likely to opt in to the research if they have an interest in violence prevention. In contrast, the American students experienced *YTM* at school and data were collected from them in the classroom. Although given the option of opting out, previous research has shown the response rates are significantly higher for studies, which require participants to opt out rather than opt in (Hunt *et al.*, 2013). Secondly, Australian data on attitudes around abusive relationships have changed substantially in recent years (Webster *et al.*, 2018) and as such even a short time difference might account for differences. Although Plourde *et al.* (Plourde *et al.*, 2016) do not state when their data collection was undertaken, some results from their longitudinal survey, which tracked students for more than 2 years were reported in Plourde *et al.* (Plourde *et al.*, 2014), suggesting their baseline data were collected no later than 2012 or at least 2 years prior to the Australian data.

A further limitation of the findings in this study concerns the method of recruitment of participants. Local organizers emailed invitations of participation for both the baseline and follow-up data to all persons who had registered for a YTM event. Neither the numbers of people invited to participate in each round of data collection, nor the numbers who attended each presentation were available to the authors. As such, it is not possible to determine the response rates. However, it is likely that invitations from local organizers, who are known to potential participants, yielded more responses than invitations sent by the authors with whom they had no prior connection would have done.

Informal feedback from local organizations suggests that there are also benefits for hosting organizations. While further research to ascertain the extent is required, there is some evidence to support the use of the *YTM* programme having built capacity among the leadership to effectively model bystander behaviours and appropriately respond to issues of violence and abuse when they occur within or affect people associated with their organization. Furthermore, as the *YTM* programme includes a panel discussion with members of local services involved in domestic violence and sexual assault, including specialist services, police, health services and counsellors, the programme is also building relationships between organizations which may not previously existed, for example between a sporting group and the regional domestic violence service.
